# Effects of Shift Work Type on Sleep Quality and Cortisol Regulation Among Nurses: A Prospective Observational Study

**DOI:** 10.3390/ijerph23050560

**Published:** 2026-04-26

**Authors:** Željka Dujmić, Štefica Mikšić, Željko Mudri, Marija Barišić, Ivana Barać, Jasenka Vujanić, Maja Čebohin, Zvjezdana Gvozdanović, Stana Pačarić, Valentina Grnja, Josip Samardžić, Ivica Mihaljević, Nikolina Farčić

**Affiliations:** 1 Faculty of Dental Medicine and Health Osijek, Josip Juraj Strossmayer University of Osijek, 31000 Osijek, Croatia; zeljka.dujmic@bolnicasb.hr (Ž.D.); smiksic@fdmz.hr (Š.M.); zmudri@fdmz.hr (Ž.M.); mbarisic@fdmz.hr (M.B.); ibarac@fdmz.hr (I.B.); jvujanic@fdmz.hr (J.V.); mcebohin@fdmz.hr (M.Č.); zvjezdana.gvozdanovic@obnasice.hr (Z.G.); spacaric@fdmz.hr (S.P.); vgrnja@fdmz.hr (V.G.); josip.samardzic@gmail.com (J.S.); 2 Faculty of Medicine, Josip Juraj Strossmayer University of Osijek, 31000 Osijek, Croatia; ivica.mihaljevic@mefos.hr; 3 General Hospital Dr. Josip Benčević, 35000 Slavonski Brod, Croatia; 4 General County Hospital Našice, 31500 Našice, Croatia; 5 Master’s Study Programme in Nursing, University of Applied Sciences in Bjelovar, 43000 Bjelovar, Croatia; 6 University Hospital Centre Osijek, 31000 Osijek, Croatia

**Keywords:** Fitbit Charge 3, cortisol, sleep quality, nurses, rotating shift work

## Abstract

**Highlights:**

**Public health relevance—How does this work relate to a public health issue?**

- Shift work in nursing represents a prevalent occupational exposure that may adversely affect sleep quality and the regulation of stress-related physiological processes.
- Objective assessments of sleep and cortisol enable early detection of health effects associated with shift work.

**Public health significance—Why is this work of significance to public health?**

- Impaired sleep and elevated cortisol levels may contribute to increased long-term health risks among nurses.
- The observed hormonal alterations demonstrated reversibility following adequate recovery.

**Public health implications—What are the key implications or messages for practitioners, policy makers and/or researchers in public health?**

- Ensuring sufficient recovery time and optimizing shift schedules are critical for protecting nurses’ long-term health.
- The implementation of wearable technology may enhance occupational health monitoring and inform preventive strategies.

**Abstract:**

Rotating shift work is a common occupational stressor among healthcare professionals. This study aimed to examine the impact of shift work on sleep quality and cortisol levels among nurses. A prospective observational study was conducted in Croatia in 2025, involving 140 nurses. Sleep quality was assessed using the Fitbit Charge 3 smartwatch over eight consecutive days, and blood cortisol was measured at two time points. Most nurses (98.6%) had a neutral chronotype. Nurses who work day shifts (DSN) achieved significantly higher sleep scores compared with rotating-shift nurses (RSN) (77, IQR 75–80 vs. 73, IQR 68–76; Mann–Whitney U test, *p* < 0.001). RSN also had significantly higher morning cortisol levels at the first measurement (median 431.6, IQR 351.3–496.2 vs. 355.15, IQR 254.9–434.2) nmol/L; *p* < 0.001). In the RSN group, morning cortisol levels at the first measurement were positively correlated with age and years of service, and negatively correlated with circadian rhythm. Smartwatch-based assessments demonstrated that rotating shift work is associated with poorer sleep quality and transient disruption of cortisol regulation. Notably, cortisol levels in rotating-shift nurses returned to levels comparable to those of DSN after two nights of rest, indicating substantial reversibility of the hormonal response. These findings highlight the short-term physiological impact of shift work and underscore the importance of optimizing work schedules and implementing strategies to reduce long-term health risks.

## 1. Introduction

Rotating shift work is essential for around-the-clock healthcare. However, it is one of the most significant occupational stressors for healthcare professionals. While it ensures uninterrupted medical care, such schedules often affect physiological, psychological, and cognitive functioning [1,2,3]. The continuous presence of nurses is critical to hospital workflow, but this model disrupts the internal circadian clock.

The circadian rhythm, or the body’s internal clock, regulates numerous physiological functions, including sleep–wake cycles, hormone secretion, body temperature, and metabolic processes. When this rhythm is disrupted, a range of consequences may arise from acute sleep disturbances to chronic health conditions such as cardiovascular disease, metabolic syndrome (a cluster of conditions increasing heart disease risk), obesity, type 2 diabetes, and depression [4,5]. Among healthcare professionals working shifts, particularly night shifts, reductions in total sleep time and decreases in both subjective and objective sleep quality are frequently observed [6,7].

Sleep disturbances are closely linked to imbalances in stress hormones, particularly cortisol. Cortisol, produced by the adrenal cortex, controls homeostasis and regulates metabolism, immunity, and cognition. Its secretion follows a daily rhythm: highest in the morning (cortisol awakening response, CAR) and decreasing throughout the day [8]. Rotating shifts can disrupt this, causing abnormal cortisol levels, altered rhythms, and blunted CAR. These changes can raise stress, lower resilience, and impair cognition [9].

Chronotype denotes stable inter-individual preferences for the timing of sleep and daily activity, typically classified into morning, intermediate, and evening types. Chronotype reflects whether a person naturally prefers to be active earlier or later in the day. It represents an important determinant of shift-work tolerance [10]. Evidence indicates that chronotype interacts with specific shift schedules to influence sleep quality: morning types generally experience reduced sleep quality following night shifts, evening types show the greatest difficulties during day shifts, while intermediate types tend to exhibit the highest adaptability across different shift patterns [10].

Traditionally, research on sleep quality and hormonal regulation among shift workers has relied primarily on subjective assessment methods, such as questionnaires and sleep diaries. Although these tools can provide valuable insights into perceived sleep quality, they are prone to bias and do not offer objective physiological data [11].

In the past decade, objective monitoring methods, including laboratory-based hormone analyses and wearable technologies, have been increasingly implemented. Wearable devices, such as commercial smartwatches, use sensors for accelerometry (measuring movement), photoplethysmography (PPG, a technique that measures blood flow), and other biometric signals to estimate sleep duration and architecture, heart rate variability, body temperature, and physical activity levels [12]. Although limitations in the accuracy of classifying specific sleep stages remain compared with the gold standard, polysomnography (PSG, a comprehensive sleep study involving various physiological measurements), most devices show strong agreement with PSG measurements when assessing total sleep time [13,14]. In the context of shift work, such devices offer a unique opportunity for continuous, non-invasive monitoring of physiological changes over extended periods and under real-world working conditions [13,14].

Previous studies in nursing populations have largely relied on subjective questionnaires to assess sleep quality [15], while objective monitoring using wearable devices remains limited [16]. Although several investigations have examined cortisol levels in relation to shift work [17,18], no research to date has simultaneously combined smartwatch-based objective sleep assessment with blood-based cortisol measurement. This study addresses this gap by integrating both physiological and wearable-derived indicators to better describe the impact of shift work on sleep and stress regulation among nurses. Part of the dataset used in the present study has been previously analyzed in a published study focusing on the comparison between subjective and objective sleep quality in nurses working different shift schedules. However, the aim of the current manuscript is substantially different. While the previous study focused exclusively on sleep quality, the present study extends the analysis by incorporating additional variables, namely chronotype (assessed using the Morningness–Eveningness Questionnaire) and cortisol levels as indicators of circadian preference and physiological stress response. Therefore, this study provides novel insights into the interaction between shift work, objective sleep parameters, chronotype, and endocrine response, including the short-term dynamics and reversibility of cortisol levels. These aspects were not addressed in the previous publication and represent a new analytical perspective.

The aim of this study was to examine the impact of shift work on sleep quality and cortisol levels among nurses, using a smartwatch for continuous sleep monitoring and laboratory blood analyses at two time points.

## 2. Materials and Methods

### 2.1. The Study Design

A prospective study was conducted from February to July 2025.

### 2.2. Participants

**Figure 1:**
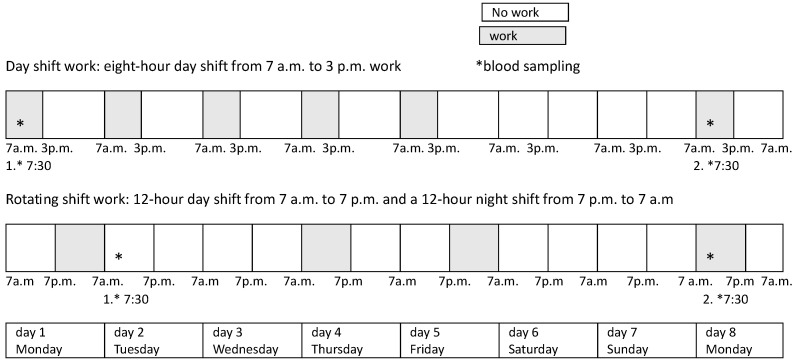
Nurses’ work schedule during the measurement period in relation to shift type. Adapted from our previous work [19], with the addition of blood sampling time points for cortisol analysis.

The study included nurses working at the General Hospital Dr. Josip Benčević in Slavonski Brod. The study included 140 nurses across all educational levels, who were divided into two groups: those working rotating shifts (RSN) and those working exclusively daytime hours (DSN). Sleep quality was monitored over eight consecutive days using a wearable smart device that objectively recorded sleep parameters. Although rotating shifts in routine clinical practice at this hospital are often irregular and variable, a standardized rotating-shift protocol was applied for the purposes of this study. To ensure consistent and comparable working conditions, this protocol was implemented during the study period and for the two preceding months, including a limited number of consecutive night shifts and a mandatory minimum of two rest days following each night shift. The day-shift group worked a fixed morning schedule (7 a.m.–3 p.m.) from Monday to Friday, with weekends off. The rotating-shift group followed a pattern of a 12-h day shift (7 a.m.–7 p.m.) and a 12-h night shift (7 p.m.–7 a.m.), each followed by two days of rest. Figure 1 illustrates the nurses’ work schedule during the measurement period in relation to shift type, including the timing of blood sampling for cortisol assessment. Nurses working rotating shifts and those working day shifts accumulated the same total number of working hours during the measurement period (48 h), despite differences in shift length and the number of working days (Figure 1).

Inclusion criteria were: nurses employed in clinical departments who provide direct patient care, have at least two years of professional experience, hold a permanent employment position, and voluntarily agree to participate in the study.

Exclusion criteria: diagnosed adrenal gland disorders, diagnosed sleep disorders, regular use of sleep medications, nephrotic syndrome, liver cirrhosis, or hypoalbuminemia of other causes. In addition, participants receiving corticosteroid therapy for any underlying disease or condition, as well as those using hormonal therapy, including oral contraceptives, were also excluded from the study.

The structured questionnaire collected demographic information, including age, gender, years of work experience, education level, shift work status, body weight, and height. In addition, data on diagnosed sleep disorders and current therapy use were obtained to assess eligibility and apply the exclusion criteria.

For the study, statistical methods were applied to calculate the appropriate sample size as follows: to detect a medium effect (d = 0.5) in the difference in numerical variables between two independent groups, with a significance level of 0.05 and a power of 0.80, the minimum required sample size was 128 participants.

This study is based on a dataset collected as part of a broader research project investigating the effects of shift work on nurses. A portion of the sleep-related data has been previously published, focusing on subjective and objective sleep quality [19]. In contrast, the present analysis includes additional variables that were not examined in the previous study, specifically chronotype assessed using the Morningness–Eveningness Questionnaire and cortisol levels measured at two time points. Furthermore, the current study explores the associations between nurse characteristics, circadian rhythm (chronotype), sleep parameters, and cortisol levels using Spearman’s correlation analysis, separately for day-shift and rotating-shift nurses. The focus of this analysis is on the physiological (endocrine) response to shift work and its short-term reversibility, which represents a distinct research objective compared with the previous publication.

### 2.3. Ethical Considerations

The study was approved by the Ethics Committee of the Dr. Josip Benčević General Hospital, Slavonski Brod (protocol code 040000/24-37, approval date: 10 June 2024), and conducted in accordance with the principles of the Declaration of Helsinki. Prior to participation, nurses were provided with an information consent form, which they signed to indicate their agreement to participate in the study. Additionally, nurses received both written and verbal explanations of the study’s objectives and expectations. The study was conducted anonymously, and nurses’ identities were not recorded. Each nurse was assigned a unique, easily memorable code to ensure confidentiality and to enable linkage of data across repeated measurements. Participation was entirely voluntary, and nurses were informed that they could withdraw from the study at any time.

### 2.4. Instruments

#### 2.4.1. Morningness–Eveningness Questionnaire (MEQ)

The Morningness–Eveningness Questionnaire (MEQ) [20] is a 19-item self-assessment questionnaire that measures an individual’s chronotype, i.e., their preference for activity and alertness in the morning or evening. Items are rated on a 4 to 5-point scale, depending on the question. The sum of all responses provides a total score ranging from 16 to 86, with higher scores indicating a stronger morning orientation (“morningness”) and lower scores indicating a stronger evening orientation (“eveningness”). Based on the total score, participants can be categorized into the following types: Definitely evening type: 16–30, Moderately evening type: 31–41, Intermediate (neutral) type: 42–58, Moderately morning type: 59–69, Definitely morning type: 70–86. The questionnaire demonstrates good reliability, with Cronbach’s α ranging from approximately 0.70 to 0.85 across different studies [21]. A Croatian-translated version of the MEQ was used [22].

#### 2.4.2. Sleep Monitoring Using the Fitbit Charge 3 Smartwatch

In this study, the Fitbit Charge 3 smartwatch (Fitbit, Inc., San Francisco, CA, USA) was used [23,24,25,26]. This is an advanced fitness device capable of continuous monitoring throughout the day and week (24/7). It tracks heart rate, exercise, oxygen saturation, all sleep stages, step count, distance, active minutes, calorie expenditure, and targeted workout modes. By connecting to a computer application, data can be exported in PDF or Excel formats. The watch evaluates overall sleep quality daily and weekly by summing individual scores for sleep duration and sleep quality. Sleep duration refers to the time spent asleep and in wakefulness periods; longer sleep corresponds to a higher score. Sleep quality includes the duration of deep and REM sleep; longer periods of both result in higher scores. Restoration is assessed by pulse rate during sleep and relaxation; elevated pulse and restless sleep lower the overall sleep quality score. All sleep events (sleep bouts) automatically detected by the Fitbit Charge 3, including both nighttime and daytime sleep, were combined and included in the analysis. Daytime naps were incorporated into the total sleep duration and overall sleep quality scores as calculated by the device’s algorithm. The maximum possible score is 100 points. Based on Fitbit’s general cut-off values, sleep scores were classified as poor (<60), moderate (60–79), good (80–89), or excellent (90–100) [23,24,25,26]. Nurses wore the smartwatches for eight days. Participants were instructed to wear the Fitbit Charge 3 smartwatch continuously on their non-dominant wrist for eight consecutive days and nights, including during sleep, removing it only when necessary (e.g., bathing or swimming). To minimize data loss and ensure data quality, participants were instructed to charge the smartwatch once daily during periods when it was not worn (e.g., while showering). Immediately after charging, they were asked to reapply the device and ensure a secure fit, enabling continuous and accurate recording of sleep and activity throughout the monitoring period.

#### 2.4.3. Laboratory Sampling—Cortisol

An Abbott Alinity i analyzer was used [27]—an advanced CMIA (chemiluminescent microparticle immunoassay) analyzer with paramagnetic microparticles and a chemiluminescent signal, for the quantitative determination of hormones, proteins, antibodies, vitamins, and other analytes. Test: Alinity i Cortisol Reagent Kit (Abbott, IL, USA). Sample type: serum. Principle: CMIA—competitive immunoassay [28].

Reference values (Abbott): Morning cortisol (08:00–10:00): 6.2–19.4 µg/dL (≈171–535 nmol/L); Afternoon cortisol (16:00–18:00): 2.3–11.9 µg/dL (≈63–328 nmol/L).

Blood samples were collected at two time points. For the rotating-shift nurses (RSN), the first sample was drawn at 07:30 following a night shift, and the second sample was collected at 07:30 after two consecutive days off, i.e., after completing two shifts in the rotation (12-h work/24-h rest/12-h work/48-h rest). For the day-shift nurses (DSN), the first blood draw was at 07:30, and the second draw was performed at 07:30 on the eighth day after the first sampling, after two consecutive days off (Figure 1).

### 2.5. Data Analysis

Categorical variables are presented as absolute and relative frequencies. Differences between categorical variables were assessed using Fisher’s exact test. The normality of continuous variables was evaluated with the Shapiro–Wilk test. Continuous data are summarized as medians and interquartile ranges (IQR). Differences in continuous variables between rotating-shift and day-shift nurses were analyzed using the Mann–Whitney U test, with Hodges–Lehmann median differences and 95% confidence intervals reported [29]. Associations between variables were assessed using Spearman’s rank correlation coefficient (rho). All *p*-values were two-sided, and statistical significance was set at α = 0.05. The statistical program MedCalc^®^ Statistical Software version 22.023 (MedCalc Software Ltd., Ostend, Belgium; https://www.medcalc.org; 2024, accessed on 25 August 2025) was used for statistical analysis.

## 3. Results

A subset of these findings has been adapted from our previous work [19]; however, this study provides new analysis examining sleep quality and cortisol levels in shift-working nurses. The study was conducted on 140 nurses, of whom 25 (17.9%) were men and 115 (82.1%) were women; 70 nurses (50%) worked rotating shifts. Regarding years of professional experience, the largest proportion of nurses, 34 individuals (24.3%), had more than 30 years of service. According to the Morningness–Eveningness Questionnaire, most nurses (138, 98.6%) were classified as intermediate (neutral) types (score range 42–58), meaning they do not show a strong preference for either morning or evening activities and have a more flexible daily routine (Table 1).

Subjective sleep quality was assessed based on nurses’ responses to the question of how they would rate their sleep over the past month. A total of 120 nurses (85.7%) reported sleeping very well, whereas two nurses (1.4%) reported very poor sleep over the past month. There was no significant difference in subjective sleep quality with respect to the type of shift work (Fisher’s exact test, *p* = 0.17). Based on the sleep score data from the smartwatch, most nurses (104, 74.3%) had an average sleep score. When examining the distribution of sleep-score categories, rotating shift work was significantly associated with poorer objectively measured sleep quality (Fisher’s exact test, *p* < 0.001) (Table 2).

The type of shift work was not significantly associated with nurses’ morning or evening chronotype. There were no significant differences in subjective sleep quality across shift work types. Nurses who work day shifts exhibited significantly better sleep parameters measured by the smartwatch compared with those who work rotating shifts (a subset of these findings has been reported previously: total sleep time, deep sleep, light sleep, REM phase [19]) and had a higher sleep score (77 vs. 73) (Mann–Whitney U test, *p* < 0.001). Rotating-shift nurses were significantly younger compared with day-shift nurses (median 40 years vs. 45 years) (Mann–Whitney U test, *p* = 0.04), whereas there were no significant differences in body mass index according to work type (Table 3).

Among rotating-shift nurses, cortisol values at the first measurement were significantly higher compared with day-shift nurses (median 431.6 vs. 355.15 nmol/L) (Mann–Whitney U test, *p* < 0.001). Additionally, the difference between the first and second measurements was significantly greater in rotating-shift nurses (Wilcoxon test, *p* < 0.001), whereas no statistically significant difference was observed at the first measurement; that is, cortisol levels increased significantly in rotating-shift nurses (Table 4).

Among day-shift nurses, age was significantly negatively correlated with deep sleep duration (Rho = −0.249), whereas this association was not observed among rotating-shift nurses. Furthermore, only among rotating-shift nurses did REM sleep show a positive correlation with years of professional experience (Rho = 0.242). Regarding cortisol levels, in the day-shift group, a significant negative correlation was found between body mass index and morning cortisol levels at both the first (Rho = −0.302) and second (Rho = −0.288) measurements.

Among rotating-shift nurses, morning cortisol values at the first measurement were positively associated with age and years of professional experience (both Rho = 0.259) and negatively and significantly associated with nurses’ circadian rhythm (Rho = −0.287); that is, higher MEQ scores (indicating a stronger morning preference) were associated with lower cortisol levels (Table 5 and Table 6).

## 4. Discussion

The results of this study show that nurses working rotating shifts have significantly poorer sleep parameters, as measured by a smartwatch, compared with nurses working day shifts. Additionally, rotating-shift nurses (RSN) exhibited significantly higher morning cortisol levels than day-shift nurses (DSN). After two nights of rest, cortisol levels in RSN were comparable to those in DSN, indicating substantial reversibility of the hormonal response and suggesting that the body strives to preserve its fundamental physiological rhythmicity despite circadian disruption. In our sample, 96.4% nurses subjectively rated their sleep as good or very good, suggesting a clear positive bias and adaptation to long-term sleep disturbances. These findings suggest that nurses may rely on cognitive and emotional coping mechanisms to perceive their sleep as adequate, despite objective evidence of reduced sleep duration, particularly among those working night shifts.

Using a smartwatch, this study found that none of the nurses working exclusively daytime schedules exhibited poor sleep quality according to the device criteria, whereas approximately 9% of RSNs did. Moreover, only 10% of RSN achieved “good” sleep quality, compared with 33% in the daytime group, while the majority of DSN showed only moderate sleep quality. These findings align with those of Feng et al. [30], who monitored 113 nurses using wearable sensors over a 10-week period. That study reported lower sleep efficiency and greater sleep fragmentation among rotating shift workers, findings consistent with ours and further underscoring the pervasive nature of this effect, irrespective of work system or geographic setting. They also report poor subjective sleep quality more frequently, which contrasts with our findings, in which most nurses rated their sleep as good or very good. Such differences may be attributable to a more favorable shift structure in our study population: although nurses worked 12-h shifts, these included only one night shift followed by two days of rest, which may support better perceived sleep quality. It should also be considered that healthcare professionals may underreport sleep difficulties, either to maintain a sense of professional competence or due to adaptation to chronic fatigue, which can influence their perception and reporting of sleep quality. The study by Galasso et al. showed that nurses working night or rotating shifts had a weaker rest–activity rhythm and a later peak in daily activity than day-shift nurses, based on 5 days of actigraphy. These findings indicate that shift work disrupts normal circadian patterns, even over a short period. The results highlight the importance of scheduling practices and workplace strategies that help reduce circadian misalignment and support better sleep and recovery in rotating-shift nurses [31]. A study that monitored hospital nurses’ sleep over six consecutive days using the Fitbit Charge 3 found notably poorer sleep outcomes among those who worked three or more consecutive night shifts, indicating that both the frequency and length of night-shift sequences further deteriorate sleep quality [32]. Extended night and rotating shift work disrupt circadian rhythms, reduce sleep quality, and contribute to cumulative negative effects on nurses [33]. Notably, the prevalence of poor sleep quality remains high even among those who no longer work night shifts [34].

The results of this study show that RSN had significantly higher morning cortisol levels immediately after a night shift (431.6 nmol/L) than DNS (355.2 nmol/L). This finding suggests an acute stress response and hyperactivation of the HPA axis, likely triggered by workload and prolonged wakefulness during a period when the body would naturally be expected to rest. Cortisol levels vary with the time of day, reflecting an underlying circadian rhythm that drives high morning concentrations, followed by a progressive decline throughout the day [35]. Most previous studies, such as that by Burek et al. [36], measured cortisol after daytime sleep and reported a reduced CAR in shift workers, reflecting chronic circadian desynchronization. Because our sampling was performed at the end of the night shift, prior to sleep, it reflects a distinct phase of the physiological stress response. This methodological difference likely accounts for the elevated, rather than decreased, cortisol concentrations observed in our study. Additionally, the study by Ljevak et al. conducted in Bosnia and Herzegovina, which used the same approach to morning cortisol sampling after a night shift, showed that nurses working irregular rotating shifts, particularly those with greater family responsibilities and longer work experience, were more likely to experience sleep disturbances between shifts. Rotating-shift nurses also had significantly higher cortisol levels than day-shift nurses, indicating increased physiological stress [37]. In our study, after two nights of rest, cortisol levels in the RSN decreased to 332.6 nmol/L, which is equivalent to the levels observed in the DSN group (332.3 nmol/L). A reduction of nearly 100 nmol/L within 48 h indicates substantial reversibility of the hormonal response. A similar recovery was reported by Andreadi et al. [38], who found that a short rest period was sufficient to temporarily normalize the circadian cortisol profile, although the authors caution that chronic exposure to night work can lead to lasting changes. Boivin et al. noted that night-shift workers who sleep during the day undergo a degree of circadian desynchronization comparable to that experienced by travelers rapidly crossing several time zones [39].

Altered cortisol patterns, such as a blunted CAR or a flattened diurnal rhythm, indicate circadian misalignment due to night-shift work. Morning values remain highest, but the difference relative to evening levels is reduced, suggesting chronic adaptation and a diminished circadian rhythm amplitude [40], as reflected in our findings. Kudielka et al. [41] reported that night-shift workers displayed significantly lower cortisol levels upon waking (who typically awaken in the afternoon, when endogenous cortisol levels are already in the descending phase of the diurnal cycle) compared to day-shift workers, accompanied by higher levels of self-reported stress and fatigue. Niu et al. [42] showed that after consecutive night shifts, cortisol secretion patterns in nurses returned to levels comparable to those of day shift workers only by the second day off. Their findings suggest that rotating-shift nurses may need at least four days to fully adjust their circadian cortisol rhythms, and that more than two days off should be allowed when switching from night to day shifts. These results highlight that adequate recovery time between shift cycles is crucial for reestablishing normal diurnal cortisol patterns and may inform scheduling strategies aimed at minimizing physiological disruptions caused by night work. Similar results have been reported in other studies [18]. In their literature review, Grosser et al. [43] reported that rotating shift work, particularly night shifts, significantly disrupts cortisol levels, the diurnal cortisol rhythm, and the CAR. Irregular shift patterns cause even greater disturbances than regular schedules. However, substantial methodological inconsistencies across studies make it difficult to determine how cortisol rhythms shift, adapt, or recover in response to night-shift work [43]. According to the systematic review by Inchingolo et al., quantitative findings indicate that optimized shift scheduling can substantially enhance staff well-being, with improvements in sleep quality ranging from 15% to 40%, underscoring the importance of carefully structured shift systems in mitigating the adverse effects of shift work [44].

The results of this study indicate that, with respect to cortisol levels, a negative and significant association was found between body mass index and morning cortisol concentrations in the DSN group. This suggests that individuals with a higher body mass index exhibit a blunted morning cortisol rise, which may reflect chronic cortisol dysregulation associated with chronic stress [8], a finding that is not unexpected in a sample of nurses. Following prolonged overload, the system responds less robustly, and morning cortisol may be lower than expected [8]. Occupational stress represents a critical issue for nurses worldwide, as the nursing profession is inherently demanding and exposes practitioners to a wide range of psychosocial and environmental risk factors [45].

The results of this study indicate that, among RSN, morning cortisol values at the first measurement were positively associated with age and years of service. The capacity to obtain uninterrupted, sufficiently long sleep declines with age, even under ideal conditions. In the study by Ljevak et al., shift workers with more years of professional experience reported greater sleep disturbances [37]. In addition, ageing is associated with a natural shift toward morningness, making night work increasingly difficult and potentially contributing to withdrawal from night shifts. In contrast, the meta-analysis by Chang and Peng indicates that nurses working rotating and fixed night shifts experience poorer sleep quality than those working fixed day shifts. Importantly, age moderated this relationship only in rotating shift work, with younger nurses (≤40 years) showing a greater decline in sleep quality than day-shift nurses, and older nurses (>40 years) showing no decline. This suggests increased vulnerability to rotating shift schedules among younger nurses [46]. These age-related changes underscore the importance of considering age when designing shift schedules [44].

Other studies emphasize that it would also be advisable to take nurses’ chronotypes into account [45,47], as evening types tend to prefer night shifts, while morning types favor day shifts. Individuals with an intermediate (neutral) chronotype experience fewer sleep disturbances compared with those who have pronounced morning or evening preferences. Over a six-year follow-up period, the chronotype profile remained largely stable, although a gradual shift toward greater morningness was observed. Importantly, individuals with extreme chronotypes were more prone to circadian disruption, which adversely impacted their sleep quality [47]. Our findings further indicate that, among RSN, morning cortisol values at the first measurement were negatively and significantly associated with circadian rhythm; specifically, higher MEQ scores (reflecting a stronger morning preference) were associated with lower cortisol levels. These results are consistent with a recent study conducted among healthcare workers [48], which demonstrated that an evening chronotype is associated with poorer sleep outcomes and dysregulation of the hypothalamic–pituitary–adrenal axis. In particular, evening types exhibited a higher prevalence of insomnia, as well as a significantly attenuated cortisol awakening response and a flatter diurnal cortisol slope, indicating impaired circadian regulation [48]. In contrast, morningness was identified as a protective factor against insomnia. Taken together, these findings support the role of chronotype as an important modifier of both sleep quality and cortisol dynamics in shift-working populations. Accordingly, consideration of chronotype in shift scheduling and the development of targeted interventions may help reduce circadian misalignment and improve health outcomes among rotating-shift nurses [38].

The findings of the study by Shiffer et al. suggest that, from an occupational health perspective, clockwise shift rotation (following the sequence “morning → afternoon → night → two days off”), which includes short and regular breaks between shifts, represents a more favorable scheduling approach compared with the sequence “afternoon → morning → morning → night → three days off,” which involves irregular breaks between shifts. Clockwise rotation is associated with longer, higher-quality sleep and improved work–life balance [49]. Although our study did not specifically examine different directions of shift rotation, these findings are relevant in the broader context of shift-work organization. The comparison between fixed day-shift nurses and rotating-shift nurses in our study highlights the impact of work schedules on circadian rhythm and cortisol dynamics, particularly in the presence of irregular breaks between shifts, which may further exacerbate circadian misalignment. Therefore, evidence supporting more favorable scheduling patterns, such as forward (clockwise) rotation with regular recovery periods, provides an important framework for interpreting our results and underscores the need to optimize shift design to mitigate circadian disruption and improve nurses’ well-being. Clockwise rotation aligns more favorably with circadian physiology. These findings underscore the importance of implementing forward-rotating schedules as a strategy to promote occupational health and enhance the sustainability of shift-work systems in nursing [49]. Although rotating shifts in routine clinical practice are often irregular, this study applied a standardized and structured schedule to ensure comparable working conditions. During the study period and the preceding two months, rotating-shift nurses worked under a protocol that limited consecutive night shifts and ensured at least two rest days following night duty. Despite this structured organization, rotating-shift nurses still demonstrated poorer sleep quality and transient cortisol disruption compared with day-shift nurses. However, the observed recovery of cortisol levels after two nights of rest suggests that adequate recovery time may substantially mitigate acute physiological effects. These findings indicate that, beyond the mere presence of night work, the structure and regularity of shift scheduling play a key role in circadian stability and occupational health outcomes.

Compared with previous research, this study offers several distinct advantages. The specific shift schedule (12–24–12–48) enables the assessment of the effects of planned recovery periods on physiological parameters, which is uncommon in studies employing standard or fixed shift patterns. The dual cortisol measurements allow for monitoring and comparing both the acute effects (immediately after the night shift) and short-term reversibility (after rest). Continuous sleep monitoring using a wearable sensor provides objective data collected over multiple consecutive days, including both workdays and days off, whereas many earlier studies relied solely on subjective methods such as questionnaires.

This study has several limitations. First, it was conducted within a single healthcare institution, which may limit the generalizability of the findings to broader populations. Although the study provides valuable insights into the relationship between cortisol levels and sleep quality among rotating-shift and day-shift nurses, objective sleep monitoring was limited to an eight-day period, offering only a short-term snapshot rather than long-term sleep patterns or variations across different shift schedules. Additionally, lifestyle factors such as diet, caffeine consumption, physical activity, and family responsibilities were not systematically collected, although they may influence sleep behavior and could confound the observed associations.

When sleep-quality data collected by a smartwatch are combined with laboratory cortisol measurements, a more comprehensive understanding of the impact of shift work on the circadian rhythm and hormonal balance is obtained. This approach enables not only a more precise assessment of the current state but also the development of individualized interventions to improve sleep quality, optimize work schedules, and prevent the long-term health consequences of shift work. Ultimately, these insights can serve as a foundation for shaping organizational policies and preventive strategies that support the health of healthcare workers and help maintain a high standard of care delivery.

Future research should adopt multicenter, longitudinal designs with extended objective sleep monitoring to better capture long-term circadian and hormonal adaptations to shift work. Additionally, incorporating lifestyle and psychosocial factors and evaluating personalized interventions based on chronotype and professional characteristics may support the development of targeted preventive strategies for healthcare workers.

## 5. Conclusions

Most nurses exhibited a neutral chronotype, and rotating-shift work was associated with poorer objectively measured sleep quality compared with day-shift work. Higher morning cortisol levels observed after night shifts suggest a temporary disruption in cortisol regulation and circadian rhythmicity, while the decrease following rest days may indicate short-term recovery. In rotating-shift nurses, cortisol levels were associated with age, years of professional experience, and chronotype, indicating that individual characteristics may influence physiological responses to shift work. The findings also suggest that, beyond the presence of night work, the structure and regularity of shift scheduling may contribute to circadian stability and occupational health outcomes. However, given the observational design, single-center setting, and short monitoring period, the results should be interpreted with caution. Further studies are warranted to confirm these findings and to explore their long-term implications.

## Figures and Tables

**Table 1:** Participant characteristics.

Variables	Number (%) of Respondents
Gender	
Men	25 (17.9)
Women	115 (82.1)
Work schedule	
Day shifts	70 (50)
Rotating shifts	70 (50)
Length of professional experience	
0–5 years	30 (21.4)
6–10 years	12 (8.6)
11–20 years	31 (22.1)
21–30 years	33 (23.6)
more than 30 years	34 (24.3)
Morningness–Eveningness Questionnaire (MEQ)	
Definite morning type (70–86)	0 (0)
Moderate morning type (59–69)	2 (1.4)
Intermediate (neutral) type (42–58)	138 (98.6)
Moderate evening type (31–41)	0 (0)
Definite evening type (16–30)	0 (0)

**Table 2:** Subjective assessment of sleep quality and smartwatch-based sleep scores in relation to shift work type.

	Number (%) of Respondents			*p* *
	DSN Group	RSN Group	Total (%)	
Subjective assessment of sleep quality				
Very bad (3)	2 (2.8)	0 (0)	2 (1.4)	0.17
Bad (2)	3 (4)	0 (0)	3 (2.1)	
Good (1)	7 (10)	8 (11)	15 (10.7)	
Very good (0)	58 (82.8)	62 (88.5)	120 (85.7)	
Smartwatch sleep score values				
Poor sleep quality	0	6 (9)	6 (4)	<0.001
Moderate sleep quality	47 (67)	57 (81)	104 (74)	
Good sleep quality	23 (33)	7 (10)	30 (21)	

* Fisher’s exact test; DSN—day-shift nurses group; RSN—rotating-shift nurses group.

**Table 3:** MEQ scores, subjective sleep quality assessments, smartwatch “sleep score” values, and participants’ age and body mass index in relation to shift work type.

	MEQ Scores	Subjective Sleep Quality Rating	Smartwatch Sleep Score Values	Age (Years)	Body Mass Index (kg/m^2^)
DSN group Median (IQR)	52 (48–54)	0 (0–0)	77 (75–80)	45 (38–51)	25.51 (23.72–30.10)
RSN group Median (IQR)	51 (50–54)	0 (0–0)	73 (68–76)	40 (26–48)	24.8 (22.47–27.68)
Difference	0	0	−5	−4	−0.85
95% CI (difference)	−1 to 1	0 to 0	−7 to −3	−10 to 0	−2.26 to 0.48
*p* *	0.84	0.27	<0.001	0.04	0.21

* Mann–Whitney U Test (Hodges–Lehmann median difference); DSN—day-shift nurses group; RSN—rotating-shift nurses group.

**Table 4:** Comparison of cortisol levels between day-shift and rotating-shift nurses.

	Cortisol (nmol/L)		Difference (First vs. Second)	95% CI (Difference)	*p* ‡
	First Measurement	Second Measurement			
DSN group Median (IQR)	355.15 (254.9–434.2)	332.35 (283.2–397.2)	−17.85	−36.6 to 1.4	0.07
RSN group Median (IQR)	431.6 (351.3–496.2)	332.55 (261.7–411.7)	−98.05	−119.7 to −78	<0.001
Difference	87.35	−4.8			
95% CI (difference)	48.69 to 126.2	−40.9 to 33.3			
*p* †	<0.001	0.82			

† Mann–Whitney U Test (Hodges–Lehmann median difference); ‡ Wilcoxon Test; DSN—day-shift nurses group; RSN—rotating-shift nurses group.

**Table 5:** Associations between nurse characteristics, circadian rhythm, sleep measures, and cortisol levels in day-shift nurses.

	Spearman’s Correlation Coefficient Rho (*p* Value)			
DSN Group	Age	Years of Experience	Body Mass Index	Nurses’ Circadian Rhythm (MEQ)
Smartwatch monitoring				
Total sleep time	−0.112 (0.35)	−0.068 (0.58)	0.115 (0.34)	−0.051 (0.68)
Sleep score	−0.221 (0.07)	−0.119 (0.33)	−0.084 (0.49)	−0.029 (0.81)
Deep sleep	−0.249 (0.04)	−0.182 (0.13)	0.026 (0.83)	−0.16 (0.19)
Light sleep	−0.009 (0.94)	0.022 (0.86)	0.098 (0.42)	0.095 (0.43)
REM phase	−0.047 (0.70)	−0.053 (0.66)	−0.007 (0.96)	−0.038 (0.76)
Number of steps	−0.216 (0.07)	−0.150 (0.22)	−0.096 (0.43)	−0.107 (0.38)
Cortisol level				
First measurement	−0.107 (0.38)	−0.066 (0.59)	−0.302 (0.01)	0.029 (0.81)
Second measurement	−0.132 (0.28)	−0.144 (0.23)	−0.288 (0.02)	−0.002 (0.99)

DSN—day-shift nurses group.

**Table 6:** Associations between nurse characteristics, circadian rhythm, sleep monitoring, and cortisol levels in rotating-shift nurses.

Spearman’s Correlation Coefficient Rho (*p* Value)				
RSN Group	Age	Years of Experience	Body Mass Index	Nurses’ Circadian Rhythm (MEQ)
Smartwatch monitoring				
Total sleep time	−0.085 (0.49)	−0.116 (0.34)	0.059 (0.63)	−0.134 (0.27)
Sleep score	−0.016 (0.90)	−0.008 (0.95)	−0.029 (0.81)	−0.084 (0.49)
Deep sleep	−0.228 (0.06)	−0.165 (0.17)	0.074 (0.54)	−0.019 (0.87)
Light sleep	0.095 (0.44)	0.025 (0.84)	−0.002 (0.99)	−0.079 (0.51)
REM phase	0.237 (0.05)	0.242 (0.04)	0.180 (0.14)	−0.035 (0.77)
Number of steps	−0.210 (0.08)	−0.107 (0.38)	0.018 (0.88)	0.057 (0.64)
Cortisol Level				
First measurement	0.259 (0.03)	0.259 (0.03)	−0.137 (0.26)	−0.287 (0.02)
Second measurement	0.038 (0.76)	0.065 (0.59)	−0.189 (0.12)	−0.103 (0.40)

RSN—rotating-shift nurses group.

## Data Availability

Data will be made available upon request by emailing the first author.

## Abbreviations

The following abbreviations are used in this manuscript:

RSN	Rotating-shift nurses
DSN	Day-shift nurses
CAR	The cortisol awakening response
PPG	Photoplethysmography
PSG	Polysomnography
MEQ	Morningness–Eveningness Questionnaire
CMIA	Chemiluminescent microparticle immunoassay
IQR	Interquartile range
Rho	Spearman’s rank correlation coefficient
